# Supplementary material for: Storage Temperature Affects Platelet Activation and Degranulation in Response to Stimuli
Source: Int J Mol Sci. 2025 Mar 24;26(7):2944. doi: 10.3390/ijms26072944 (PMC11989061; doi:10.3390/ijms26072944)
Supplement: Supplementary file 1 [file ijms-26-02944-s001.zip › ijms-3497405-supplementary.pdf]

International Journal of *Molecular Sciences*

MDPI

Appendix

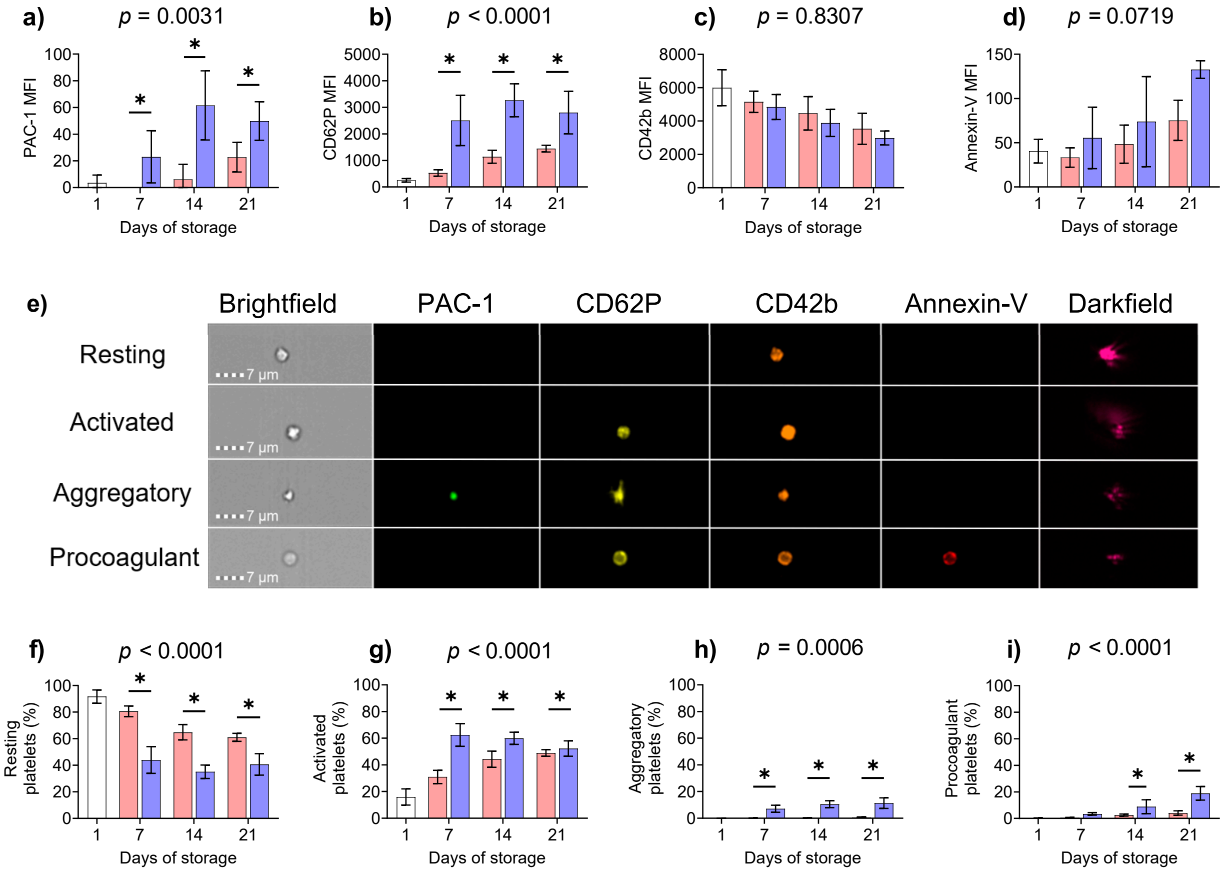

Figure S1. Cold storage affects the surface receptor phenotype of platelets. Platelets were sampled on day 1 (baseline;) or following room-temperature ( ) or cold storage ( ) on day 7, 14 and 21 post-collection. Platelets were stained with PAC-1-FITC, CD62P-PE, CD42b-PE-Dazzle 594 and annexin-V-APC and analysed by imaging flow cytometry at 60x magnification. Platelet events were gated based on size (area and aspect ratio) and scatter (darkfield). The median fluorescent intensity (MFI) of **a)** PAC-1, **b)** CD62P, **c)** CD42b and **d)** annexin-V were examined following the acquisition of 7500 platelet events. **e)** Representative brightfield and fluorescence images and the proportion of platelets in each subpopulation are shown for **f)** resting (PAC-1-/CD62P-/CD42b+/Annexin-V-), **g)** activated (PAC-1-/CD62P+/CD42b+/Annexin-V-), **h)** aggregatory (PAC-1+/CD62P+/CD42b+/Annexin-V-) and **i)** procoagulant (PAC-1-/CD62P+/CD42b+/Annexin-V+) subpopulations. Data represent the mean ± standard deviation (error bars, n=6). Significance was determined by two-way ANOVA comparing the effects of temperature (RT vs 4 °C) on platelet samples over time, with the interaction p-value presented. * = p<0.05 compared to room-temperature at the same time-point.

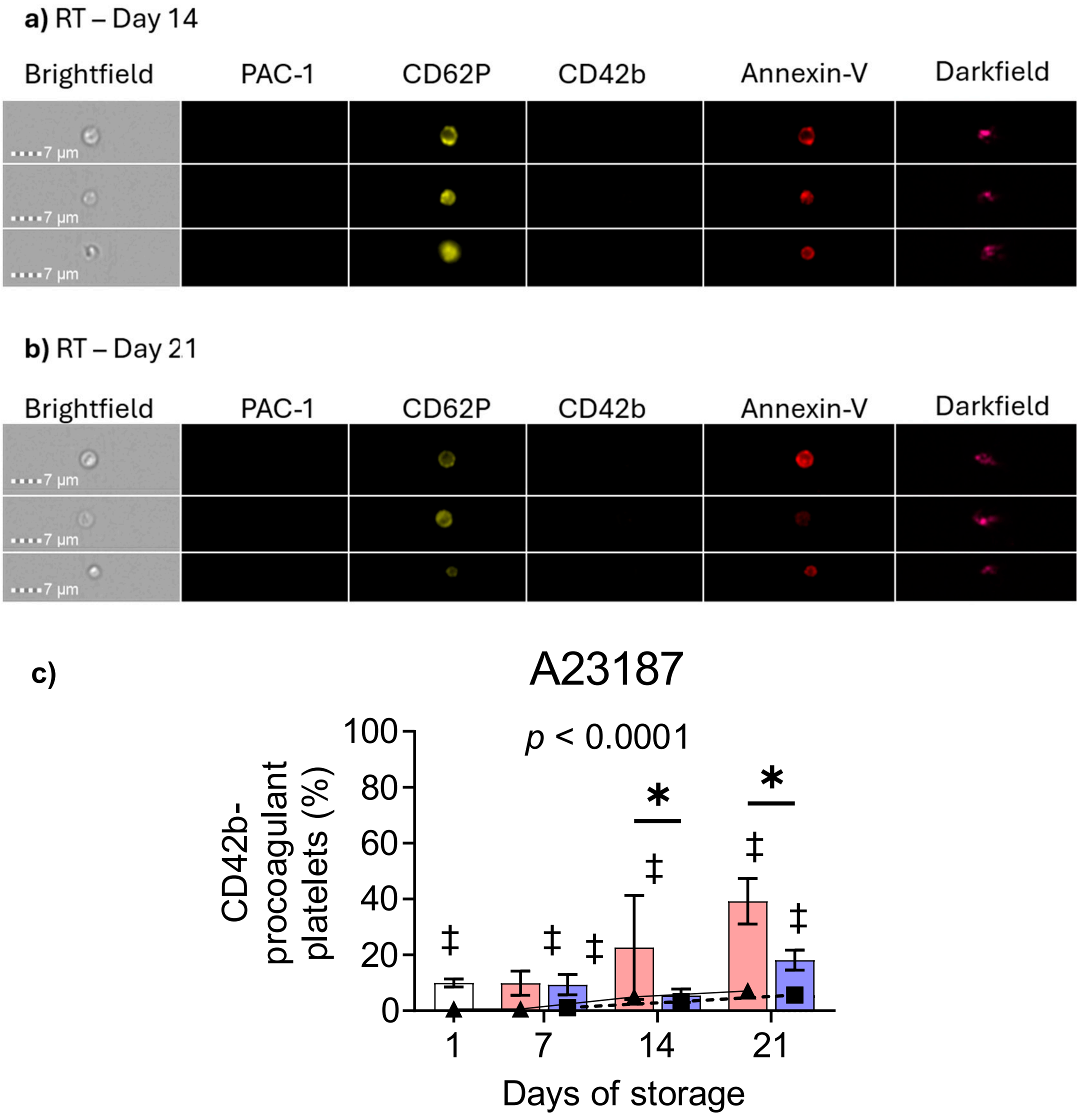

Figure S2. A proportion of room-temperature stored platelets do not exhibit CD42b binding following activation with A23187. Platelets were sampled on day 1 (baseline;) or following room-temperature ( ) or cold storage ( ) on day 7, 14 and 21 post-collection. . Platelets were stained with PAC-1-FITC, CD62P-PE, CD42b-PE-Dazzle 594 and annexin-V-APC and analysed by imaging flow cytometry at 60x magnification. Platelet events were gated based on size (area and aspect ratio) and scatter (darkfield). Representative brightfield and fluorescence images are shown of a PAC-1-/CD42b-/CD62P+/Annexin-V+ subpopulation of platelets observed following stimulation with A23187 in room-temperature samples at **a)** day 14 and **b)** 21 of storage. The percentage of PAC-1/CD42b-/CD62P+/Annexin-V+ platelets present in **c)** unstimulated and following activation with A23187 (10 µM) is shown over storage. Data represent the mean + standard deviation (error bars, n=6). The baseline percentage of CD42b- procoagulant platelets from **a)** unstimulated samples, at RT ( ) or 4°C ( ), has been overlaid on the corresponding **b) - e)** stimulated samples for comparisonSignificance was determined by two-way ANOVA comparing the effects of temperature (RT vs 4 °C) and stimulation on platelet samples over time, with the interaction p-value presented. * = p<0.05 compared to room-temperature at the same time-point. ‡ = p<0.05 compared to unstimulated RT or 4 °C platelets at the same time-point.

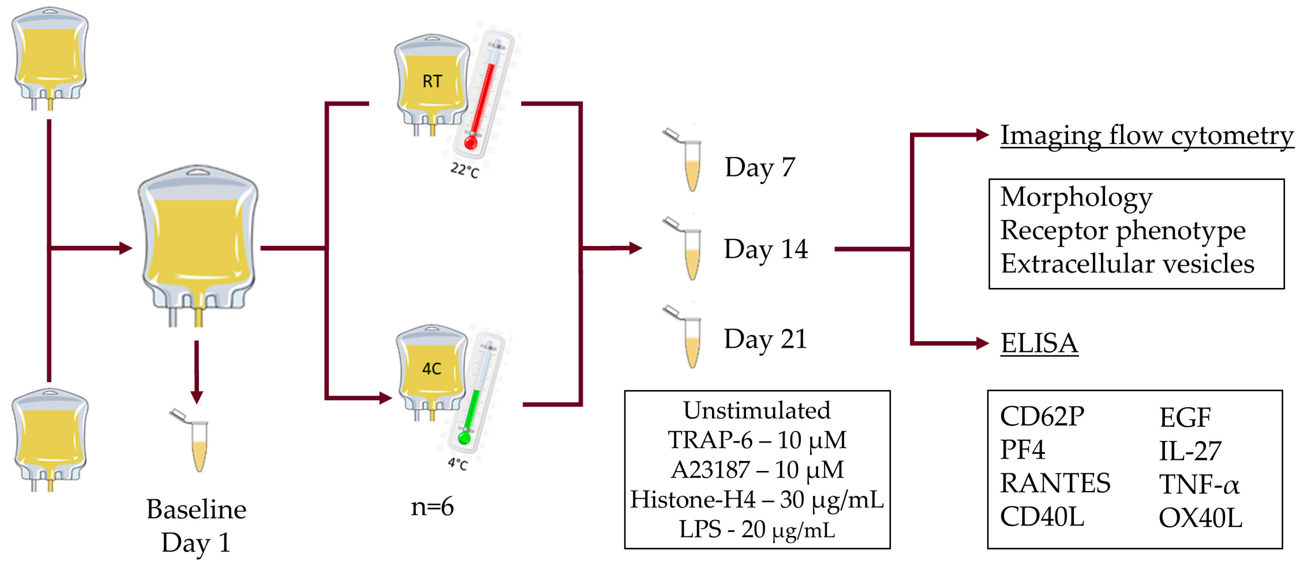

Figure S3. Study design and the sampling schedule for stored platelet concentrates.

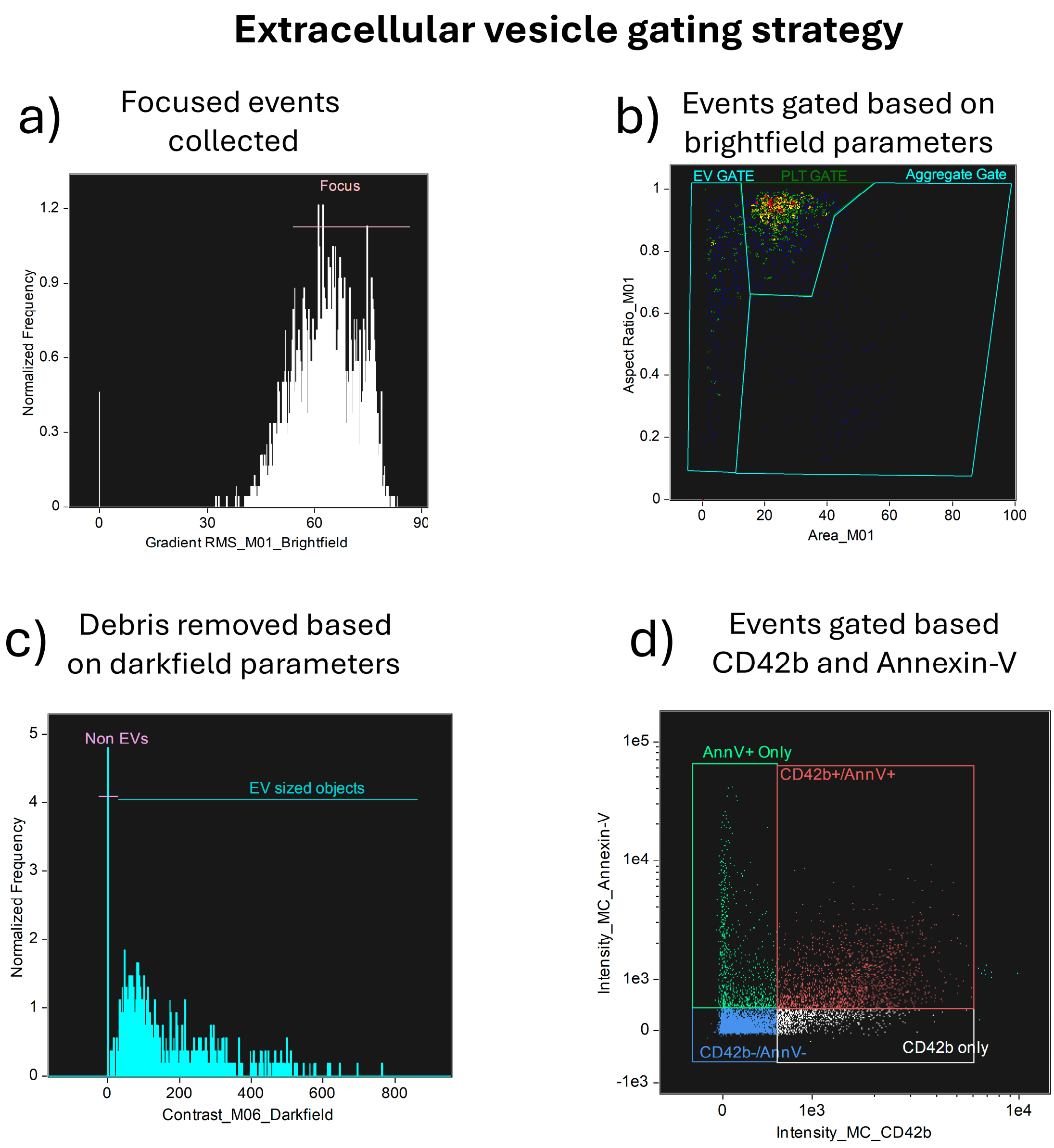

Figure S4. Gating strategy used to measure and phenotype platelet derived extracellular vesicles by imaging flow cytometry. PAC 1 FITC, CD62P-PE, CD42b-PE-Dazzle 594 and annexin-V-APC and analysed by imaging flow cytometry at 60x magnification equipped with one charge coupled device (CCD) camera, two excitation lasers (488 nm: 100 mW, 642 nm: 150 mW) and a side scatter laser (785 nm: 2 mW). Following sample collection the data files were imported into IDEAS v6.2 software and compensated prior to analysis. **a)** Focused events were selected for analysis based on brightfield (Gradient RMS_M01) values. **b)** The focused events gated based on known size limits of platelets and extracellular vesicles with reference to control beads. **c)** Small debris were separated from likely extracellular vesicles based on darkfield (scatter) parameters (Contrast_M06). **d)** Extracellular vesicles which stained dual positive for CD42b and annexin-V were classified as phosphatidylserine positive platelet derived extracellular vesicles.
